# Editorial: Highlights From TERMIS EU 2019

**DOI:** 10.3389/fbioe.2020.604661

**Published:** 2020-11-04

**Authors:** Maria Chatzinikolaidou, Dimitrios I. Zeugolis

**Affiliations:** ^1^Department of Materials Science and Technology, University of Crete, Crete, Greece; ^2^Institute of Electronic Structure and Laser, Foundation for Research and Technology-Hellas, Crete, Greece; ^3^Regenerative, Modular & Developmental Engineering Laboratory, National University of Ireland Galway, Galway, Ireland; ^4^Science Foundation Ireland, Centre for Research in Medical Devices, National University of Ireland Galway, Galway, Ireland; ^5^Regenerative, Modular & Developmental Engineering Laboratory, Faculty of Biomedical Sciences, Università della Svizzera Italiana, Lugano, Switzerland

**Keywords:** tissue engineering, regenerative medicine, cell therapies, functional biomaterials, preclinical models

Tissue Engineering and Regenerative Medicine International Society (TERMIS) is the most prominent organization in the field of tissue engineering and regenerative medicine globally. TERMIS promotes education, research, innovation, clinical translation, and social responsibility within the field of tissue engineering and regenerative medicine through regular meetings, training courses, scientific and lay publications, outreach activities, and other forms of communication. TERMIS provides an international forum for informed discussion on the challenges and achievements of tissue engineering therapies.

This special issue is associated with the TERMIS EU Chapter 2019 meeting at Rhodes, Greece, 27-31 May. The theme of the meeting was “*Tissue Engineering Therapies: From concept to clinical translation and commercialisation.”* This special issue covers all aspects of tissue engineering and regenerative medicine, including computational models for the cost efficient production of cell-based tissue engineered products (Mehrian et al.); development of media [e.g., sodium hyaluronate supplemented culture media for chondrogenic differentiation (Monaco et al.)] and tools [e.g., non-invasive techniques to measure oxygen gradients in cell culture (Peniche Silva et al.), processes to generate and culture three dimensional cell spheroids in a microfluidic set-up (Lopa et al.), cell-derived matrices to control cell phenotype (Penolazzi et al.)] for more accurate cell culture and for effectively monitoring the fate of implantable devices [e.g., multiparametric optical bioimaging (Elagin et al.)]; the potential of large animal models in regenerative medicine (Ribitsch et al.); and clinical indication specific discussion [e.g., inflammation and bone repair (Goodman et al.), electrospun scaffolds for cartilage engineering (Yilmaz and Zeugolis)].

Advancements in chemistry and engineering have made numerous new materials available and nano/micro fabrication technologies have potential in modern tissue engineering and regenerative medicine. With this in mind, this special issue discusses a range of natural and synthetic systems [e.g., fibrin-agarose hydrogels (Campos et al.), gellan gum hydrogels (Gomes et al.), elastin-like recombinamer hydrogels (Ibáñez-Fonseca et al.), poly(ethylene terephthalate) electrospun scaffolds decorated with poly(lactic acid-co-glycolic acid) (PLGA) or poly(glycolic acid) (PGA) electrosprayed nanoparticles (McCormick et al.), tripolyphosphate crosslinked chitosan/gelatin biocomposite inks (Fischetti et al.), inorganic calcium phosphate, chitosan, and hyaluronic acid scaffolds (Rammal et al.), polycaprolactone-polypyrrole printed conductive biomaterials (Vijayavenkataraman et al.)] for a diverse range of clinical targets, as well as advances in stimuli responsive materials as controlled delivery vehicles (Laurano and Boffito; Boffito et al.).

Considering that cells are the basic building blocks of tissues (both healthy and diseased), contemporary tissue engineering and regenerative medicine exploits the power of cells for the development of implantable devices that will revolutionize healthcare due to their therapeutic and reparative capabilities (e.g., hybrid bioprinting of chondrogenically induced human mesenchymal stem cell spheroids with a photocrosslinkable methacrylamide-modified gelatin printed medium (De Moor et al.), cornea cells with a plastic compressed nanostructured fibrin-agarose biomaterial (Garzón et al.), vascular endothelial growth factor (VEGF)-transduced bone marrow stem cells with a hydroxyapatite scaffold (Largo et al.), human pluripotent stem cell-smooth muscle cell-endothelial cell vascular organoids embedded in collagen/fibrinogen/fibronectin matrices (Markou et al.), but also for the development of organ on chip models to study cell functions [e.g., osteoblast maturation toward osteocytes and matrix mineralisation (Nasello et al.), aged tendon organoids (Yan et al.)] and of *in vitro* pathophysiology models for drug screening purposes [e.g., skin (Schmidt et al.; Zurina et al.), tumor (Gupta et al.), intervertebral degenerative disc (Li et al.) models].

Tissue engineering and regenerative medicine have the potential to provide effective treatments for uncurable injuries and diseases, potentially enabling the development of *in vitro* models for the study of disease progression and to assess the potential of novel therapies. This collection of manuscripts captures the state-of-play in this field, indicating that the development of effective therapies is underway.

## Author Contributions

All authors listed have made a substantial, direct and intellectual contribution to the work, and approved it for publication.

## Conflict of Interest

The authors declare that the research was conducted in the absence of any commercial or financial relationships that could be construed as a potential conflict of interest.

